# Does low parental warmth and monitoring predict disordered eating in Australian female and male adolescents?

**DOI:** 10.1186/2050-2974-2-S1-O29

**Published:** 2014-11-24

**Authors:** Isabel Krug, Anisha Sorabji, Ross King, Primrose Letcher, Craig Olsson

**Affiliations:** 1 The University of Melbourne, Melbourne, Australia; 2 School of Psychology, Deakin University, Melbourne, Australia; 3 Murdoch Children’s Research Institute, Melbourne, Australia; 4 The University of Melbourne (Paediatrics and Psychological Sciences), Melbourne, Australia; 5 The Royal Children's Hospital, Melbourne, Australia

## Objectives

To investigate the predictive power of low parental warmth and monitoring at age 13-14 years and their individual, combined and interactive effects on disordered eating (DE) outcomes at age 15-16 years.

## Method

Participants included 1391 (684 females) adolescents and their parents. The parents completed data on parenting practices and the adolescents provided data on the drive for thinness, body dissatisfaction and bulimia EDI subscales.

## Results

Low monitoring in girls was significantly associated with bulimia, whereas for boys none of the individual parenting styles were significantly related to any of the EDI subscales. For females, exposure to both low warmth and monitoring was associated with a 4.5,5.8 and 6.5 fold increase in the odds of reporting body dissatisfaction, drive for thinness and bulimia, respectively [80%,74% and 59% of which was attributable to the additive interaction of both parental risk factors respectively]. For males, exposure to both low warmth and monitoring was associated with a 2.5 fold increase in the odds of bulimic behaviour,41% of which was attributable to the joint action of both parenting risk factors.

## Conclusions

Whereas for females a neglectful-disengaging parenting style revealed a range of DE symptoms, for boys such a parenting style was predictive mainly of bulimic behaviours.

This abstract was presented in the **Parental Roles in Prevention and Support** stream of the 2014 ANZAED Conference.

